# Enzyme Activity of Natural Products on Cytochrome P450

**DOI:** 10.3390/molecules27020515

**Published:** 2022-01-14

**Authors:** Hua-Li Zuo, Hsi-Yuan Huang, Yang-Chi-Dung Lin, Xiao-Xuan Cai, Xiang-Jun Kong, Dai-Lin Luo, Yu-Heng Zhou, Hsien-Da Huang

**Affiliations:** 1 School of Life and Health Sciences, The Chinese University of Hong Kong, Shenzhen, Shenzhen 518172, China; zuohuali@cuhk.edu.cn (H.-L.Z.); huanghsiyuan@cuhk.edu.cn (H.-Y.H.); yangchidung@cuhk.edu.cn (Y.-C.-D.L.); xiaoxuancai@link.cuhk.edu.cn (X.-X.C.); dailinluo@link.cuhk.edu.cn (D.-L.L.); 119010482@link.cuhk.edu.cn (Y.-H.Z.); 2 Warshel Institute for Computational Biology, The Chinese University of Hong Kong, Shenzhen, Shenzhen 518172, China; 3 School of Computer Science and Technology, University of Science and Technology of China, Hefei 230027, China; 4 State Key Laboratory of Quality Research in Chinese Medicine, Institute of Chinese Medical Sciences, University of Macau, Macao 999078, China; hangkxj@163.com

**Keywords:** natural product, herbal ingredient, cytochrome P450, herb–herb interaction, herb–drug interaction, combinatorial synergism

## Abstract

Drug-metabolizing enzymes, particularly the cytochrome P450 (CYP450) monooxygenases, play a pivotal role in pharmacokinetics. CYP450 enzymes can be affected by various xenobiotic substrates, which will eventually be responsible for most metabolism-based herb–herb or herb–drug interactions, usually involving competition with another drug for the same enzyme binding site. Compounds from herbal or natural products are involved in many scenarios in the context of such interactions. These interactions are decisive both in drug discovery regarding the synergistic effects, and drug application regarding unwanted side effects. Herein, this review was conducted as a comprehensive compilation of the effects of herbal ingredients on CYP450 enzymes. Nearly 500 publications reporting botanicals’ effects on CYP450s were collected and analyzed. The countries focusing on this topic were summarized, the identified herbal ingredients affecting enzyme activity of CYP450s, as well as methods identifying the inhibitory/inducing effects were reviewed. Inhibitory effects of botanicals on CYP450 enzymes may contribute to synergistic effects, such as herbal formulae/prescriptions, or lead to therapeutic failure, or even increase concentrations of conventional medicines causing serious adverse events. Conducting this review may help in metabolism-based drug combination discovery, and in the evaluation of the safety profile of natural products used therapeutically.

## 1. Introduction

Absorption, distribution, metabolism, and excretion (ADME) is a pretty complex process that a drug will go through. Notably, drug metabolism plays a pivotal role in determining the fate of drugs. Herein, in drug research and development, pharmacokinetics research must be conducted to identify drugs’ transformed forms and properties in the body. Drug-metabolizing enzymes (DMEs) can convert drugs/compounds to metabolites with different biological effects and are critical in determining the bio-availability and effectiveness of orally administered drugs [1]. The cytochrome P450 (CYP450) enzyme family is one of the most vital DEMs, has been found in many tissues, e.g., liver, intestine, lung, heart, and brain [2,3], and is mainly distributed in the liver and intestine, namely hepatic and intestinal/enteric CYP450s. The CYP450 catalyzes the phase I metabolism of conventional drugs. The activity of CYP450s could also be inhibited or induced by various xenobiotics (e.g., herbal ingredients), which will eventually be responsible for the most metabolism-based herb–herb or herb–drug interactions. Compounds from herbal or natural products are frequently involved in such interactions.

Though the traditional usage of herbal medicine is mainly documented in Asia, particularly in China, South Korea, India, and Japan, such complementary medical therapies have been applied extensively over the last decade. These interactions are decisive both in drug discovery regarding the synergistic effects, and drug application regarding unwanted side effects. The concerns of their effects on CYP450s mainly focus on two aspects. On the one hand, from the drug development point of view, herbal medicines are often prescribed in combinations, namely herbal formulae, to enhance efficacy and reduce toxicity [4,5]. Partially due to the affected CYP450s, clearance of the effective compounds may retard, and the risk of exposure to the toxic ingredients may decrease. On the other hand, from the perspective of herb–drug interaction (HDI), herbal constituents can act as inducers of CYP450s, thereby increasing the rate of metabolism of the drug to result in therapeutic failure; besides, herbal compounds can act as inhibitors of CYP450s as well, resulting in the reduced activity of CYP450s, thereby enhancing the therapeutic effects by increasing the concentration of the co-used drug. Since plant-derived products are often self-prescribed either alone or in combination with over-the-counter medicines, and integrated herbal therapies with Western medicine are considered to exert better efficacy in clinical. Therefore, herbal products are noteworthy facts that may affect drug metabolism by modulating CYP450s.

Currently, the clinical influence caused by inhibiting or inducing effects of various xenobiotics on DEMs gained increasing attention. Pelkonen et al. have comprehensively summarized the inhibitors and inducers, including pharmaceuticals and herbal/botanical natural products, of the specific CYP450s in humans in 2008 [6] and updated in 2020 [7]. Mukherjee et al. [8] reviewed the botanicals as medicinal products and their effects on DEMs in 2011. Besides, Zhu et al. [9] recently developed a database named INTEDE, comprehensively collating the interactions on DEMs of drugs approved by the U.S. FDA, investigational drugs, or xenobiotics, including herbal ingredients. Since CYP450s act as one of the most significant DEMs.

Herbal ingredients could affect CYP450 enzymes at multi-levels, including the expression of mRNA [10,11] or protein [12,13], and enzyme activity [14,15,16]. This review mainly focuses on the herbal products’ modulation of enzyme activity. Identifying the specific subtype provides the basis for the compatibility of herbal formulae and integrated herbal medicine with Western medicine, as well as interactions between drugs and natural products. We conducted this review as a comprehensive compilation of the effects of herbal ingredients on CYP450 enzymes compared to conventional pharmaceutics to develop a broader understanding of the pharmacological/clinical implication of interactions on CYP450s, to provide the basis for compatibility and rational administration in clinical practice.

## 2. Results and Discussion

### 2.1. Search Results, Study Inclusion

**Figure 1:**
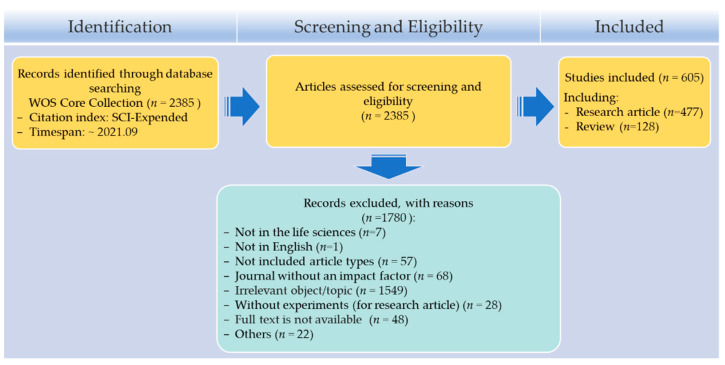
PRISMA flow diagram detailing the number of papers included at each stage and the reasons for removal. Please see Supplementary File S1 for detail.

The literature survey and screening resulted in a total of 605 full-text studies for inclusion in this review (Figure 1; Supplementary File S1). In total, 2385 items were identified by searching the WOS, after removing the papers that were not concluded in the life sciences (*n* = 7), we read the abstracts of 2378 articles for relevance. After deeper examination, we further excluded 1780 articles for reasons including the papers published in the journal without an impact factor (*n* = 68); these papers were published neither as research articles nor as review articles (*n* = 57); their topics were not relevant to the effects of natural products on the enzyme activity of CYP450s (*n* = 1549), the results were stated in research articles without experimental validation (*n* = 28), and some other reasons (*n* = 22). These without available full text were excluded as well (*n* = 48).

### 2.2. Study Characteristics

**Figure 2:**
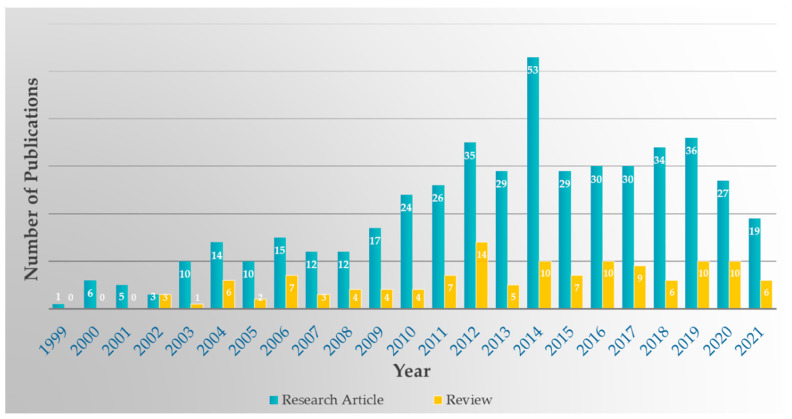
Publications (research articles and reviews) reporting the activity of natural products on CYP450s.

The majority (477, ~79.0%) of the papers included were research papers, and the rest were reviews (128, ~21.0%). This emerging topic is gaining more and more attention in the scientific community worldwide, particularly in the last decade, as witnessed by the notable increase in the number of research articles published in peer-reviewed journals in this field, as shown in Figure 2.

Historically, herbal medicines have been used due to traditional and cultural beliefs, and their usage continues even nowadays. Particularly in recent years, the worldwide recognition that natural products have gained and the crucial role of naturally derived products have played in drug discovery [17,18] promote the concern of the effects that the herbal products may induce on CYP450s.

**Figure 3:**
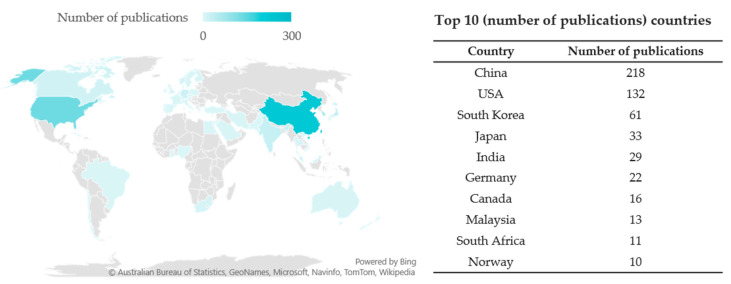
Geographical coverage of papers reporting the activity of natural products on CYP450s, and the top 10 countries ranked by the number of papers.

When analyzing countries by their number of papers on this topic, China was the most productive country, followed by the United States (USA), South Korea, Japan, India, Germany, etc., as shown in Figure 3, the geographical coverage of papers reporting the activity of natural products on CYP450s, and the top 10 countries ranked by the number of papers. Though this topic attracted the eyes of a wide range of countries, the majority of publications were contributed by the Asia countries (especially China) and the USA.

Undoubtedly, natural products catch more attention in Asia due to their historical prevalence and clinical benefits, while the view of natural products in Western countries is more delicate than in Asia due to the potential clinical risks, though approximately 25% of currently commercialized medications are derived from plants or traditional medicine [18]. Due to the loose regulatory requirements of herbal medicines and possible self-prescribed remedies, there are still multiple concerns on co-administrated herbs and conventional drugs, like potential herb–drug interaction-based side effects or therapeutic failure, particularly in the field of cardiovascular therapy [19]. However, no matter how much or little herbal products are accepted as recommended clinical medication outside Asia, there is a consensus that both in the development of small molecular drugs and research on the herbal medicines, their CYP mediated metabolism and their modulation on CYP450s are all crucial and essential [20,21].

The data show that natural products’ inhibition and induction activity on CYP450 enzymes gradually obtains a deep concern in the time and geographic span. In the case of the compounds/constituents/extracts interacting, their activities on CYP450s have been reported to be clinically relevant. To avoid unwanted side effects of the patient taking them and the benefit of drug discovery, we further reviewed the current methodologies in detecting the action and the reported typical affections on the major sub-type CYP450s accounting for drug metabolism, from a large amount of literature that documents the modulation effects of natural products on the CYP450 enzyme activity.

### 2.3. Methods for Detecting CYP450 Enzyme Activity

For new drug research and development (R&D), in January 2020, the FDA updated two guidelines from the 2017 draft of in vitro and clinical drug–drug interaction (DDI) guidance, entitled “In vitro Drug Interaction Studies—Cytochrome P450 Enzyme—and Transporter-Mediated Drug Interactions Guidance for Industry” and “Clinical Drug Interaction Studies—Cytochrome P450 Enzyme—and Transporter-Mediated Drug Interactions Guidance for Industry” [22]. The guidelines for in vitro investigation recommend conducting the essential studies to evaluate the potential for metabolism-mediated drug interactions, including (1) determining if the investigational drug is a substrate of metabolizing enzymes; (2) determining if the investigational drug is an inhibitor of metabolizing enzymes in both a reversible manner (i.e., reversible inhibition) and time-dependent manner (i.e., time-dependent inhibition (TDI)); (3) determining if the investigational drug is an inducer of metabolizing enzymes [23]. In addition, the clinical guideline aims at (1) determining whether the investigational drug alters the pharmacokinetics of other drugs; (2) determining whether other drugs alter the pharmacokinetics of the investigational drug; (3) determining the magnitude of changes in pharmacokinetic parameters; (4) determining the clinical significance of the observed or expected DDIs; (5) informing the appropriate management and prevention strategies for clinically significant DDIs [24].

These guidelines for new drug R&D are also directly related to research on natural products. Currently, to detect the activity of natural products on CYP450s mainly via in vitro and in vivo experiments (in rats/mice, or heather volunteers, namely clinical trials). The in vitro investigation allows a preliminary detection of the activity on CYP450s of natural products.

#### 2.3.1. In Vitro

In in vitro testing, different models can be used to evaluate the inhibitory or inducing effects on cytochromes: human/rat liver microsomes [25,26], human hepatocytes, and recombinant human CYP enzyme assay [27,28], human intestinal microsomes [29], human intestinal cell lines [30], etc. Furthermore, in recent years, there are some commercialized products available based on these models, e.g., Vivid^®^ CYP450 screening kits, P450-Glo™ assays, etc., were used to detect the Coptidis Rhizoma’s activity on CYP3A [31] and Bulbine natalensis’s activity on CYP3A4, CYP2C9, CYP2B6, and CYP1A2 [32,33], Stevia rebaudiana Bertoni and steviol’s activity on CYP3A4 and CYP2C9 [34], Uncaria tomentosa (Samento) and Otoba parvifolia (Banderol)’s activity on CYP3A4 and CYP2C19 [35].

CYP450s that participate in drug metabolism are mainly distributed in the liver and intestine [36]. We retrieved the method information on detecting the activity of CYP enzymes from research articles, and we found that, for evaluating the natural products’ modulation effects, over 60% of the in vitro studies were conducted on liver microsomes of humans/rats, seldom (~2%) on intestinal microsomes, indicating the emphasis routinely has been placed on hepatic events. However, as emphasized [37], enteric metabolism can arguably be as important as liver metabolism; both are determinants of orally administered drugs, especially natural products, since oral dosing is the preferred and predominant route of administration for these herbal medicines or health supplements. In oral dosing, the small intestine serves as the gateway into the systemic circulation via the provision of a biological barrier, uptake and efflux transport, and metabolic clearance. Indicating both liver- and intestinal-based experimental systems serve essential functions in assessing natural products’ affection on the enzyme activity of CYP450s.

The inhibitory effects of natural products on CYP450s are mainly divided into two modes: reversible (competitive or non-competitive) inhibition and irreversible (mechanism-based inhibition, MBI, namely suicide inhibition). The phenomenon that the metabolism of drugs by CYP450s to form reactive metabolites that bind tightly to the active site of an enzyme is referred to as MBI, leading to long-lasting irreversible inhibition of the enzymes [38]. In early 2005, Fontana et al. has summarized that the mechanism-based inhibitors have particular features, which make them recognizable by in vitro tests [39]. Herein, the in vitro assays could be utilized to test the influence of herbal xenobiotics on enzyme activity and could be mainly conducted to detect whether the inhibition is time-, concentration- and NADPH-dependent.

#### 2.3.2. In Vivo

Next, the tests conducted in vivo identify whether the tested natural products will influence the clearance of specific substrate which has been known to be metabolized by particular isoforms of CYP450s. Then, the inhibitory or inductive potential will be inferred from the reduced or increased clearance of the substrate. Among the research articles we included in the detailed analysis, nearly one-third of the research conducted in vivo experiments in rats/mice (preclinical, ~21%) and health volunteers (human clinical, ~12%).

The strength of in vivo study lies in that it considers the utmost complexity of lives and the exposure to a large number of other chemical substances through diet, environment, etc., thus making the result more reliable and indicative of clinical significance. However, the major drawback is that unlike in vitro study which has the capacity to identify the inhibitory or inductive activity directly and unveil their mechanism, in vivo studies can solely reveal the influence of natural products on the metabolism of substrate concurrently intake. Therefore, if we want to illustrate the underlying mechanism of inhibition or induction, subsequent in vitro studies will be needed.

#### 2.3.3. Probe Drug Assay

In both in vitro and in vivo investigation, the probe drug assay has been extensively applied, which analyzes the modulated enzyme activity by monitoring the concentration change of metabolites of probe drug, in the presence or absence of the tested natural products (herbal ingredients, herbal extracts, etc.) [40,41]. Furthermore, the cocktail probe assay was established based on probe assay to evaluate the modulating effects on multiple enzymes simultaneously. A cocktail approach can simultaneously evaluate a drug’s inhibition or induction potential for multiple CYP450s as long as the study is properly designed [22].

**Figure 4:**
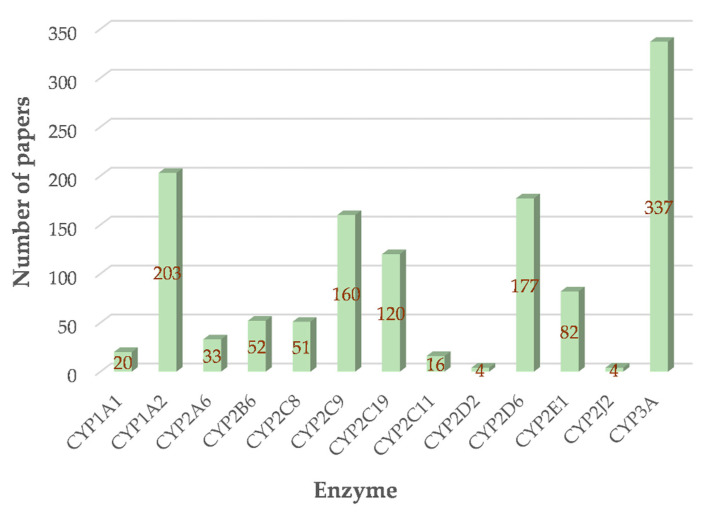
Number of papers reporting the modulation effects of natural resources on each CYP450 enzyme.

In a probe drug assay or cocktail probe assays, firstly, the CYP450s to be investigated should be determined. In the FDA guidance of in vitro and clinical studies, the isoforms of CYP450s that need to be focused on are CYP1A2, CYP2B6, CYP2C8, CYP2C9, CYP2C19, CYP2D6, and CYP3A, due to the most clinical drugs undergo biotransformation in the body through these CYP450 isozymes; thus, they are accounting for the most of potential HDI/DDI. In addition, the current studies for herbal ingredients’ modulation on CYP isoforms are also based-on CYP1 family (CYP1A1, CYP1A2), CYP2 family (CYP2A6, CYP2B6, CYP2C8, CYP2C11, CYP2D6, CYP2C9, CYP2C19, CYP2E1, CYP2J2), and CYP3 family (mainly CYP3A, including CYP3A1, CYP3A4, CYP3A5). The number of papers reporting the modulation effects of natural resources on each CYP450 enzyme was summarized. As shown in Figure 4., the CYP3A possessed the highest attention, particularly the CYP3A4 (247/337). As reported, in FDA-approved drugs, roughly 65% were substrates, 30% inhibitors, and about 5% inducers of CYP3A, and inhibition and induction of CYP3A explained most of all observed clinical interactions [7,42,43]. Based on the frequency, the CYP1A2, CYP2D6, CYP2C9, CYP2C19, CYP2E1, CYP2B6, CYP2C8 are also caught close attention. Among the ~200 research articles reporting the modulation activity of CYP1 family, more than 90% were focused on CYP1A2, which is the major hepatic member of the CYP1 family [6].

Both the FDA’s recommendation and the current frequently studied isoforms provide an overview of the isoforms concerned in existing studies, and in future work, the isoforms selected may vary depending on the property of natural products, the potential HDIs, and research aims.

Secondly, proper probe drugs need to be selected. Before the past decade, as summarized [8], the detection methods were mainly based on Spectro, fluorimetry, radiometry, and high-performance liquid chromatography (HPLC). From the information we retrieved, however, the majority of the studies detected the concentration of metabolized probes by HPLC (including HPLC-MS, UPLC-MS), which are non-optical methods. Generally, the criteria for an excellent probe substrate are high selectivity, good sensitivity, and high conversion rate (turnover), as well as commercial availability and good chemical stability for both substrate and metabolite(s) [21,44].

The detailed information of commonly used probe substrates for CYP450s (human/rat), along with the HPLC-MS methods used for their analysis, is listed in Table 1.

### 2.4. Natural Products’ Modulation on CYP450 Isoforms

**Figure 5:**
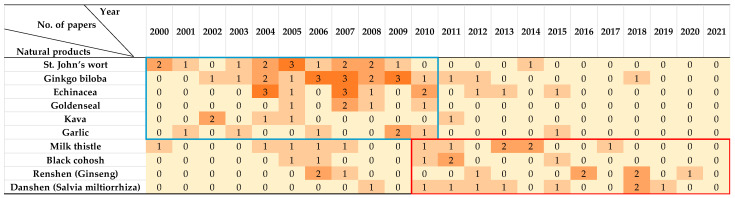
The number of research articles reporting the CYP450s’ modulation effects of the most popular natural products.

To date, the use of herbal supplements has been world-widely recognized and plays a vital role in nearly every culture, including Asia, Africa, Europe, and the Americas. In this study, we manually annotated 477 eligible papers, implying evidence is emerging that particular herbs and herbal ingredients can modulate the activity of CYP450s. The 477 papers focused on multifarious objects, vary from herbal ingredients, herbal extracts, preparations containing multiple ingredients/herbal extracts, or marketed products. Several therapeutically active dietary supplements or herbal medicines have been highly concerned. As shown in Figure 5, before the past decade, a higher number of publications reported the CYP450s’ modulation of St. John’s wort, Ginkgo biloba, Echinacea, Goldenseal, Kava, and Garlic, while in the past decade the focus of attention gradually shifted to Milk thistle, Black cohosh, Renshen (Ginseng), and Danshen (Salvia miltiorrhiza).

Multiple reviews have summarized relevant results on this topic narratively in detail [8,66,67], which have provided a wealth of content. Besides, many other reports have also reviewed specific natural products notably widely reported from the years 2000 to 2010, including their effects on CYP450, e.g., Echinacea in hepatopathy [68], from its phytochemistry, pharmacology, to safety; clinical risks of St John’s Wort co-administration [69]; interactions of Ginseng with therapeutic drugs [70].

Considering the pioneer works have provided wealth of content regarding to the effects of St. John’s wort, Ginkgo biloba, Echinacea, Goldenseal, Kava, and Garlic on CYP450s, in this study, we preferred to emphasize on the discussion of Milk thistle, Black cohosh, Renshen (Ginseng), and Danshen (Salvia miltiorrhiza), as presented in Table 2. For much more detailed annotated literatures and summaries of other natural products, please refer to Supplementary File S1.

#### 2.4.1. Milk Thistle

Milk thistle (*Silybum marianum*), known as Mary thistle and holy thistle, is a thistle of the genus Silybum, a flowering herb related to the daisy and ragweed family (Asteraceae). The plant is native to the Mediterranean regions of Europe, North Africa, and the Middle East [66]. People have traditionally used milk thistle for problems with the liver and gallbladder and therapeutic potential in diabetes [71,72].

Some in vitro studies indicated the extracts or main ingredients of Milk thistle may inhibit multiple CYP450s, i.e., CYP2C8, 2C9, 2B6, 2C19,3A4/5 (Table 2); however, the clinical study in 2014, implied that the exposure to Milk thistle extract has no significant influence on CYP1A2, CYP2C9, CYP2D6, or CYP3A4/5 activities [73], and the clinical outcome consistent with human studies that conducted more earlier [74,75,76]. To date, the reasons for the conflict of results in in vitro and in vivo studies remain unknown.

#### 2.4.2. Black Cohosh

Black cohosh (*Actaea racemose*) is a shrub-like plant native to the eastern forests of North America, and Native Americans have used it for menopausal symptoms such as hot flashes, premenstrual discomfort, and dysmenorrhea [67].

The most recent in vitro studies indicated that the Black cohosh could inhibit CYP2D6, 2C8, 2C19, and 3A4 (Table 2). The inhibition effects on 2D6 and 3A4 by 75% ethanolic extract of black cohosh are controversy from the previous reported in vitro test of commercialized black cohosh products [77] or clinical results [74]. The different results may be induced by different extract methods. Nevertheless, additional studies in humans by using extracts with essential quality control are desirable to evaluate the safety of concomitant use of black cohosh and conventional drugs.

#### 2.4.3. Renshen

Renshen (Ginseng, *Panax ginseng* Meyer) is a traditional herbal medicine used worldwide. Ginseng and Red Ginseng (the prepared products of Ginseng) are the world’s most popular herbal medicines and exhibit a wide range of pharmacologic activities [67].

The major updates of Ginseng come from the clinical trials demonstrating a negligible effect on CYP1A2, 2C9, 2C19, 2D6, 3A [78,79,80], while some other research indicated induction effect Ginseng/Red Ginseng on CYP3A [81,82]. In addition, the in vivo cocktail studies on rats showed an inhibitory effect of Sailuotong (SLT, a fixed combination of Panax ginseng, Ginkgo biloba, and Crocus sativus extracts) on CYP3A, which may be attributed to Ginseng and ginkgo cooperatively, and induction effects on CYP1A2 which may be attributed to its herbal component of Ginseng to a large extent [40].

#### 2.4.4. Danshen

Danshen derived from the roots and rhizome of *Salvia miltiorrhiza* Bge., which possesses antithrombotic properties have a long history of treating cardiovascular diseases (e.g., arteriosclerosis, ischemic heart disease, stroke) [83], and is widely used in Asia, including China, Japan, and Korea, in United States, Australia, and Holland, etc. [84].

The recent updates of Danshen’s effect on CYP450s are listed in Table 2. Except several opposite results that have been concluded from different studies (including, the induce [16] or negligible effects on CYP3A4 [85]), multiple investigations of Danshen [86] or its typical ingredients suggested an inhibition potential on CYP1A2 or/and CYP2C9. Tanshinone I, tanshinone IIA, and cryptotanshinone were potent competitive CYP1A2 inhibitors, medium competitive inhibitors of CYP2C9 [87]; Miltirone showed moderate inhibition on CYP1A2 (IC50 = 1.73 μM) and CYP2C9 (IC50 = 8.61 μM) [88]. Furthermore, these results are consistent with the results published in 2008, that tanshinone I, tanshinone IIA, and cryptotanshinone were potent competitive inhibitors of CYP1A2, danshensu was a competitive inhibitor of CYP2C9 [86]. Meanwhile, the Guanxinning injection, a marketed herbal product composed of Danshen and Chuanxiong (*Ligusticum* *chuanxiong* Hort.), showed an induced effect on CYP1A2 [89], indicating the activity of Danshen may be altered by the combinatorial effects when combined with Chuanxiong.

Unlike the Milk thistle and Black cohosh that could be used solely, Renshen and Danshen are representative herbal medicines that are frequently applied in combination with others in traditional Chinese medicines. Many studies have investigated the combinatorial rules behind the herbal pairs [90] or drug pairs [91] that mainly focus on the direct targets or genes. Inferred from the cases mentioned above of Sailuotong and Guanxinning preparation, the altered CYP modulation effects, from one aspect, might be contributed by the combinatorial synergism, while further exploration is wanted.

**Table 2:** Effects of selected natural products on CYP450s (from 2010~2020).

Natural Products	CYP450 Species	CYP450	Effects on CYP450	Method	Ref.
Milk thistle extracts and eight isolated constituents	Human	CYP3A	Inhibit (The extract silymarin and constituents … demonstrated >50% inhibition of CYP3A activity …)	In vitro (human liver and intestinal microsomes)	[29]
Milk thistle extract	Human	CYP1A2 CYP2C9 CYP2D6 CYP3A4/5	——(Exposure to milk thistle extract produced no significant influence on CYP1A2, CYP2C9, CYP2D6, or CYP3A4/5 activities.)	Clinical trial	[73]
Milk thistle extract	Human	CYP1A2 CYP2A6 CYP2B6 CYP2C8 CYP2C9 CYP2C19 CYP2D6 CYP2E1 CYP3A4	Inhibit (… the extract significantly inhibited CYP 2B6, 2C8, 2C9, 2C19, 2E1, and 3A4…) ——(but not likely, and are remote for CYPs 2C19, 2D6, and 3A4.)	In vitro (human hepatocytes and human liver microsomes), HPLC-MS	[92]
Milk thistle	Human	CYP2C9	Inhibit (The results indicated milk thistle as the most potent CYP2C9 inhibitor.)	In vitro (human liver microsomes), HPLC	[93]
Milk thistle	Human	CYP2C8	Inhibit (Isosilibinin, a mixture of the diastereoisomers isosilybin A and isosilybin B, was found to be the most potent inhibitor, followed by isosilybin B...)	In vitro (human liver microsomes), LC/MS-MS.	[94]
7-O-methylated analogues of flavonolignans from Milk thistle	Human	CYP2C9 CYP3A4/5	Inhibit (CYP2C9 activity was most sensitive to inhibition, … followed by CYP3A4/5 and …)	In vitro (human liver or intestinal microsomes), HPLC	[95]
Milk thistle aqueous/ methanolic extracts	Human	CYP2C9 CYP2B6 CYP2C19 CYP3A4	Inhibit (The present work indicates that inhibition of CYP2C9 occurs with the aqueous extracts, IC50 = 64.2 µg/mL…The methanolic extract caused significant inhibition of CYP2B6, CYP2C9, CYP2C19, and CYP3A4.)	In vitro (N-in-one cocktail), LC/MS-MS	[96]
Black cohosh	Human	CYP2D6 CYP3A4	—— (Previous in vivo studies in humans have concluded that CYP2D6 and CYP3A4 are not inhibited by black cohosh. The present data are in agreement with these findings.)	In vitro (N-in-one cocktail), LC/MS-MS	[96]
Commercial liquid (ethanol) extracts of black cohosh	Human	CYP2C19	Inhibit (one of the three most potent interactions were: Black cohosh and CYP2C19 (IC50 0.37 μg/mL).	In vitro (microplate-based assays using cDNA-expressed CYP450 isoforms and fluorogenic substrates)	[97]
75% ethanolic extract of black cohosh	Human	CYP2D6 CYP3A4	Inhibit (In vitro metabolic interactions between black cohosh and tamoxifen via inhibition of cytochromes P450 2D6 and 3A4.)	In vitro (human liver microsomes), LC-MS	[98]
Methanol extracts of garlic, echinacea, saw palmetto, valerian, black cohosh and cranberry	Human	CYP2C8	Inhibit (All herbal extracts showed inhibition of CYP2C8 activity...)	In vitro (human liver microsomes), LC/MS/MS	[99]
Red ginseng	Human	CYP2C9 CYP3A4 CYP1A2 CYP2C19 CYP2D6	——(Red ginseng poses minimal risks for clinically relevant CYP- or OATP-mediated drug interactions and is well tolerated.)	Clinical trials, Cocktail	[78]
Sailuotong (SLT), a fixed combination of Panax ginseng, Ginkgo biloba, and Crocus sativus extracts	Rat	CYP1A2 CYP3A1/2	Induce-CYP1A2 (repeated administration of SLT induced CYP1A2 by enhancing... The influence is attributed to its herbal component of ginseng to a large extent.) Inhibit- CYP3A (The inhibition of SLT on CYP3A was likely attributed to ginseng and gingko cooperatively.)	In vivo (cocktail), LC-MS/MS	[40]
Red ginseng	Human	CYP1A2 CYP2C9 CYP2C19 CYP2D6 CYP3A4	——(No significantly different drug interactions were observed between fermented red ginseng and the CYP probe substrates)	Clinical trial	[79]
Red ginseng	Human	CYP1A2 CYP2C9 CYP2C19 CYP2D6 CYP3A4	——(RG has no relevant potential to cause CYP enzyme- or P-gp-related interactions.)	Clinical trial	[80]
Panax ginseng	Human	CYP3A	Induce (Ginseng appeared to induce CYP3A activity in the liver and possibly the gastrointestinal tract.)	Clinical trial	[81]
Korean red ginseng (KRG)	Human & Mice	CYP3A CYP2D	Induce-CYP3A Inhibit-CYP2D (The area under the curve for OH-midazolam/midazolam catalysed by CYP3A was increased significantly by the administration of 2.0 g/kg KRG extract for 2 and 4 weeks. CYP3A-catalysed midazolam 1′-hydroxylation also increased significantly in a dose- and time-dependent manner…Whereas CYP2D-catalysed dextromethorphan O-deethylation decreased in a dose- and time-dependent manner in vivo.)	In vitro (human liver microsomes), in vivo, LC-MS/MS	[82]
Tanshinones of Danshen	Human	CYP1A2 CYP2C9 CYP2E1 CYP3A4 CYP1A2	Inhibit (Tanshinone I, tanshinone IIA, and cryptotanshinone were potent competitive CYP1A2 inhibitors; medium competitive inhibitors of CYP2C9; medium competitive inhibitors of CYP2E1 for tanshinone I and 10.8 μM for crytotanshinone; but weak competitive inhibitors of CYP3A4. Dihydrotanshinone was a competitive inhibitor of human CYP1A2 and CYP2C9, a noncompetitive inhibitor of CYP3A4 but an uncompetitive CYP2E1 inhibitor.)	In vitro (human Liver Microsomes), HPLC	[87]
Danshen capsules	Human	CYP3A4	Induce (The results suggested that multiple dose administration of Danshen capsules could induce cytochrome P450 (CYP) isoenzymes, thereby increasing the clearance of clopidogrel.)	Clinical trial	[16]
Danshen extract	Rat	CYP3A	—— (Orally administered Danshen had no substantial effect on the pharmacokinetics of docetaxel and clopidogrel, suggesting the negligible safety concern of Danshen in P-gp- and CYP3A-mediated interactions in vivo.)	In vivo (cocktail), LC-MS/MS	[85]
Miltirone (from Danshen)	Human	CYP1A2 CYP2C9 CYP2D6 CYP3A4	Inhibit (Miltirone showed moderate inhibition on CYP1A2 (IC50 = 1.73 μM) and CYP2C9 (IC50 = 8.61 μM), and weak inhibition on CYP2D6 (IC50 = 30.20 μM) and CYP3A4 (IC50 = 33.88 μM).)	In vitro (human liver microsomes), HPLC	[88]
Danshen components	Human	CYP2C8 CYP2J2	Inhibit (salvianolic acid A was a competitive inhibitor of CYP2C8 and mixed-type inhibitor of CYP2J2. alvianolic acid C had moderate noncompetitive and mixed-type inhibitions on CYP2C8 and CYP2J2, respectively. Tanshinone IIA was a moderate competitive inhibitor of CYP2C8. Dihydrotanshinone I had moderate noncompetitive inhibition on CYP2J2, but mechanism-based inhibition on CYP2C8. Tanshinone I was a moderate competitive inhibitor of CYP2C8.	In vitro (recombinant human CYP2C8 and CYP2J2 systems), LC-MS/MS	[100]
Danshen	Human	CYP1A2	Inhibit (CYP1A2 activity was decreased with an increasing inhibitor concentration, confirming the inhibition of caffeine metabolism in vivo.)	In vitro (human liver microsomes), clinical trials, HPLC.	[101]
Guanxinning injection (Danshen, Chuanxiong)	Rat	CYP1A2	Inhibit (The in vivo and in vitro results demonstrated that GXNI could induce CYP1A2 activity in rats.)	In vivo, in vitro, UPLC-MS/MS.	[89]
Tanshinone I, tanshinone IIA, and cryptotanshinone, baicalein, osthole, quercetin, cordycepin, and sodium tanshinone IIA sulfonate (From Danshen)	Human	CYP1A2	Inhibit (tanshinone I, tanshinone IIA, and cryptotanshinone exhibited remarkable inhibition on CYP1A2,... baicalein, osthole, quercetin, cordycepin, and sodium tanshinone IIA sulfonate showed moderate inhibition on the CYP1A2…)	In vitro (high throughput inhibitor screening kit)	[102]

## 3. Materials and Methods

### 3.1. Data Retrieval

The Web of Science^TM^ platform was used for the literature survey to achieve a dataset of studies reporting the inhibition/induction effects of natural products on CYP450. The search query was performed in the WOS core collection based on keywords: CYP, drug-metabolizing enzyme, and herbal product. Search Terms: All Fields = (CYP OR cytochrome-P450 OR drug-metabolizing-enzyme*) AND Topics = (herb* OR herbal-products OR herbal-supplements OR natural-supplement* OR botanical-supplement* OR phytotherap* OR dietary-supplementation OR plant-extract OR traditional-medicine* OR natural-product* OR botanical*), and in life/medical science focused.

### 3.2. Screening and Eligibility

As per the preferred reporting items for systematic reviews and meta-analyses (PRISMA) guidelines selection criteria [103], publications were extracted from the WOS database, and we have all the search results listed and systematically integrate and screen the listed sources, and carefully label each item the reason for including or excluding in the excel. Firstly, the duplicates are screened. Secondly, eligibility criteria were determined prior to the commencement of this review, as shown in Table 3.

### 3.3. Annotated Bibliography

The country information of the authors that contributed to each record was also retrieved to generate the data for describing the geographical coverage of papers reporting the activity of natural products on CYP450s. Due to the multi countries collaboration, an article might be contributed by authors from different countries, so in this case, each country will be counted once in this study.

After finishing the primary screening, we have all the eligible papers downloaded, and we read the abstract or whole text of each research article to decide if the source is still relevant. Then, we labeled each eligible research article by adding the information that retrieved manually, including the natural products that each paper investigated (ingredients, herbal extracts, or formulae/preparations.), the tested CYP450s, the effects and the type of effects on CYP450s, the species of CYP450s tested, the methods used, the probes or substrates if mentioned. The statistical data were then generated to describe the current status of research in this field, like the conventionally used methods in detecting such effects, the natural products that were commonly concerned, the CYP450s that are most likely to be affected. Additionally, the eligible reviews were not processed as the research articles, but they were still important to inform us of what has already been reviewed and what new knowledge we can add in this field.

## 4. Conclusions

Drug discovery involves targets’ identification/validation, and candidates’ ADMET evaluation, which remains complex, costly, and unpredictable. Good knowledge of the potential mechanisms of herbal drug interactions is necessary for assessing and minimizing clinical risks in drug R&D and may even shed light on developing synergism combinations.

CYP enzymes are crucial in metabolism and could be influenced by a wide range of xenobiotics, including natural products/herbal supplements. In this review, we systematically collected the current studies reporting the natural products’ modulation on the activity of CYP450s. Then, we summarized them from a global perspective rather than a narrative review by reporting the increasing attention in the time and geographic span, the conventional methods for detecting the modulated activity in vitro and in vivo, as well as the natural products that were most concerned. Regarding future research, it is worth noting that there are numerous investigations on regulating CYP450s’ activity that have been conducted, and the results may provide valuable references. While some limitations still exist in this study, we did not consider the research on the regulation of mRNA level or protein level, and the natural products acting as the substrate of CYP450s are not discussed either, though these processes are also crucial in affecting drug metabolism. In the future, we will look into the CYP450s-based HDI or DDI more comprehensively by taking these issues into account.

## Figures and Tables

**Table 1:** Commonly used CYP450s and their substrate in probe assays along with the HPLC-MS methods.

Enzyme	Tissue Sites	Probe	Metabolite	Ref.
CYP1A1	Liver, intestine	Phenacetin	Phenacetin O-deethylation (Acetaminophen)	[45]
		Ethoxyresorufin	Ethoxyresorufin O-de-ethylase	[46]
CYP1A2	Liver	Phenacetin	Phenacetin O-deethylation (Acetaminophen)	[47]
		Caffeine	Paraxanthine	[48,49,50]
		Methoxyresorufin	Methoxyresorufin O-demethylase	[46]
CYP2A6	Liver, lung	Coumarin	Coumarin 7-hydroxylation	[47]
		Methoxsalen	N/A	[51]
CYP2B6	Liver, lung	Bupropion	Bupropion hydroxylation	[47]
CYP2C6	Liver	Tolbutamide	N/A	[52]
CYP2C8	Liver	Paclitaxel	Paclitaxel 6-hydroxylation	[47]
CYP2C9	Liver, intestine	Diclofenac	Diclofenac 4′-hydroxylation	[47,53]
		Tolbutamide	Tolbutamide 4-hydroxylation	[54]
CYP2C19	Liver, intestine	(R)-Omeprazole	(R)-Omeprazole 5-hydroxylation	[47]
		S-Mephenytoin	S-Mephenytoin 4-hydroxylation	[55,56]
CYP2C11	Liver	S-Mephenytoin	S-Mephenytoin 4-hydroxylation	[57]
		Tolbutamide	Tolbutamide 4-hydroxylation	[49,58]
CYP2D6	Liver, intestine	Dextromethorphan	Dextromethorphan O-demethylation (dextrorphan)	[47,59]
		Bufuralol	Bufuralol 1-hydroxylation	[55]
CYP2E1	Liver, lung	Chlorzoxazone	Chlorzoxazone 6-hydroxylation	[47]
		4-Methylpyrazole	N/A	[60]
CYP3A1	Liver	Dapsone	N-acetyl dapsone	[61]
		Midazolam	Midazolam 1-hydroxylation	[62]
CYP3A4	Liver, intestine	Midazolam	Midazolam 1-hydroxylation	[47]
		Daclatasvir	N/A	[63]
		Testosterone	Testosterone 6β-hydroxylation	[21,64,65]

**Table 3:** Eligibility criteria of selected articles.

No.	Eligibility Criteria
1	Not in the life Sciences
2	Not in English
3	Not included article types. e.g., proceedings, feature, editorial material.
4	Journal without an impact factor
5	Irrelevant object/topic (The studies focusing on the regulation of gene expression or protein level were not included; the studies discussing the CYP450s that participate in the biosynthesis of bioactive natural products were not included as well. ONLY the studies that demonstrate the inhibition or induction on the activity of the CYP450 enzyme were included.)
6	Without experiments (for research article)
7	Full text not available
8	Other

## Data Availability

The data presented in this study are available in article and Supplementary File S1.

## Supplementary Materials

The following are available online, Supplementary File S1: Literature screening, eligibility and the annotated bibliography of research article.
